# Threshold responses of floating meadow fish communities to floodplain forest cover in the lower Amazon River

**DOI:** 10.1111/cobi.70110

**Published:** 2025-08-15

**Authors:** Sam Grinstead, Caroline C. Arantes, Kirk O. Winemiller, Leslie C. Kelso‐Winemiller, Jonas Alves de Oliveira, Miguel Petrere, Carlos E. C. Freitas

**Affiliations:** ^1^ School of Natural Resources and the Environment West Virginia University Morgantown West Virginia USA; ^2^ Department of Ecology and Conservation Biology Texas A&M University College Station Texas USA; ^3^ The Mamirauá Institute for Sustainable Development Tefe Brazil; ^4^ PPG ECOMAR and PPG CTA Universidade Santa Cecília Santos Brazil; ^5^ Departamento de Ciências Pesqueiras Universidade Federal do Amazonas Manaus Brazil

**Keywords:** community ecology, environmental filtering, floating meadows, floodplains, functional traits, land cover, Neotropical fish, threshold responses, cobertura terrestre, ecología de comunidades, filtrado ambiental, llanuras aluviales, peces neotropicales, praderas flotantes, rasgos funcionales, respuestas de umbral, 阈值响应, 功能性状, 环境过滤, 洪泛平原, 群落生态学, 土地覆盖, 漂浮草甸, 新热带区鱼类

## Abstract

Forest cover is positively associated with fish biomass and fisheries yield in the Amazon River floodplain, and many species enter flooded forests to feed, spawn, or seek refuge from predation. Floating macrophyte beds, known as floating meadows, in Amazon floodplains support high fish diversity and serve as nursery habitat for many fishes of high commercial importance. We surveyed fish from floating meadows in floodplain lakes along the lower Amazon River to evaluate variation in fish abundance in relation to forest cover and local environmental variables. Species associations with forest cover were estimated with threshold indicator taxa analysis (TITAN2). The analysis identified taxa that gradually increased in abundance and occurrence as forest cover increased. Many species gradually increased at approximately 40% forest cover in the local landscape. Taxa that decreased as forest cover increased exhibited thresholds, whereby their abundance and occurrence declined rapidly when forest cover exceeded approximately 9% and when it was about 20%. Small‐bodied, sedentary species with equilibrium and opportunistic life‐history strategies (i.e. functional groups) and Cichlidae and Characidae (taxonomic groups) were indicators of high forest cover, whereas large‐bodied, migratory species with periodic and intermediate life‐history strategies and Serrasalmidae were indicators of low forest cover. Our findings could be used to predict how the taxonomic and functional structure of fish communities inhabiting floating meadows would respond to deforestation.

## Abstract (es)

Respuestas de umbral de las comunidades de peces de pradera flotante a la cubierta forestal de llanura aluvial en la cuenca baja río Amazonas

**Resumen:**

La cubierta forestal está positivamente asociada con la biomasa de peces y el rendimiento de la pesca en la llanura inundable del río Amazonas, y muchas especies entran en los bosques inundados para alimentarse, desovar o buscar refugio de la depredación. Los lechos de macrófitas flotantes, conocidos como praderas flotantes, en las llanuras aluviales del Amazonas albergan una gran diversidad de peces y sirven de hábitat de cría para muchas especies de peces de gran importancia comercial. Estudiamos los peces de praderas flotantes en lagos de llanura inundable a lo largo de la cuenca baja del río Amazonas para evaluar la variación en la abundancia de peces en relación con la cubierta forestal y las variables ambientales locales. Las asociaciones de especies con la cubierta forestal se estimaron mediante el análisis de taxones indicadores de umbral (TITAN2). El análisis identificó taxones que aumentaban gradualmente en abundancia y presencia a medida que aumentaba la cubierta forestal. Muchas especies aumentaron gradualmente a partir de ~40% de cobertura forestal en el paisaje local. Los taxones que disminuían a medida que aumentaba la cubierta forestal mostraban umbrales, según los cuales su abundancia y presencia disminuían rápidamente cuando la cubierta forestal era >~9% y cuando era ~20%. Las especies sedentarias de cuerpo pequeño con estrategias vitales de equilibrio y oportunistas y los Cichlidae y Characidae (es decir, grupos funcionales) fueron indicadores de una cubierta forestal alta, mientras que las especies migratorias de cuerpo grande con estrategias vitales periódicas e intermedias y los Serrasalmidae fueron indicadores de una cubierta forestal baja. Nuestros hallazgos podrían utilizarse para predecir cómo respondería a la deforestación la estructura taxonómica y funcional de las comunidades de peces que habitan las praderas flotantes.

## 亚马逊河下游的漂浮草甸鱼类群落对洪泛平原森林覆盖的阈值响应

【摘要】

亚马逊河洪泛平原的森林覆盖与鱼类生物量及渔业产量之间存在正相关关系, 许多物种会进入被洪水淹没的森林觅食、产卵或躲避捕食者。亚马逊洪泛平原的浮水大型水生植物床(即漂浮草甸)支撑着丰富的鱼类多样性, 并为许多具有重要商业价值的鱼类提供了育幼栖息地。本研究调查了亚马逊河下游洪泛平原湖泊的漂浮草甸中的鱼类, 以评估鱼类丰度与森林覆盖及局部环境变量之间的关系。我们利用阈值指示类群分析(TITAN2)估算了物种与森林覆盖率的相关性。分析结果识别出了随森林覆盖率增加而丰度和出现频率逐渐增加的类群。许多物种在局部景观的森林覆盖率达到约40%时开始逐渐增多。随森林覆盖率增加而减少的类群则存在阈值, 即当森林覆盖率超过约9%和约为20%时, 其丰度和出现频率迅速下降。小体型、定居型、具有平衡型和机会型生活史策略的物种, 以及慈鲷科(Cichlidae)和脂鲤科(Characidae)的物种(即功能群)是高森林覆盖的指示类群, 而大体型、迁移型、具有周期型和中间型生活史策略的物种, 以及锯脂鲤科(Serrasalmidae)的物种, 则是低森林覆盖的指示类群。我们的研究结果可用于预测栖息于漂浮草甸的鱼类群落如何在类群和功能结构方面对森林砍伐做出响应。**【翻译:胡怡思;审校:聂永刚】**

## INTRODUCTION

Land‐cover changes drive shifts in ecological communities worldwide (Lambin et al., 2019; Meyer & Turner, 1992). Some ecological systems undergo rapid state transitions in response to gradual changes along a continuous environmental gradient (Groffman et al., 2006; Huggett, 2005; Toms & Lesperance, 2003). These shifts often result from changes in an environmental gradient toward less favorable conditions until population threshold responses (tipping points) occur and are indicated by a rapid decline in occurrence and abundance (Baker & King, 2010; Röpke et al., 2017; Sasaki et al., 2008). Species‐specific thresholds to environmental gradients are influenced by organismal traits and the magnitude of changes (Baker & King, 2010; Colwell & Rangel, 2009; Swift & Hannon, 2010; Turner, 1989). For example, ecological generalists may thrive in response to human‐driven land‐cover changes, whereas specialists may be adversely affected or extirpated (Kupfer & Franklin, 2009). Analysis of species‐level or functional‐group responses allows for estimation of thresholds for major community shifts in response to environmental change (Baker & King, 2010; Brejão et al., 2017; Chen & Olden, 2020).

Improved understanding of ecological responses to environmental gradients is needed to address worldwide changes in land cover; river–floodplain systems are a particular priority (Chen & Olden, 2020; Martins et al., 2021; Wang et al., 2003, 1997). River floodplains are among the planet's most productive and biodiverse ecosystems, particularly in tropical regions, where high biodiversity is produced by spatial and temporal heterogeneity in environmental conditions, such as the seasonal flood pulse and environmental gradients (Correa et al., 2022; Junk et al., 1989; Ward et al., 1999). In the lower Amazon River floodplain, agriculture‐driven deforestation has caused a 50% decline in forest cover since 1970 (Renó et al., 2011). The loss of floodplain forest cover threatens Amazonian fish conservation because these forests provide critical habitat during the seasonal flood pulse when river waters inundate the forest (Correa & Winemiller, 2018; Correa et al., 2022; Goulding, 1980, 1993). Deforestation significantly reduces fish biomass and fisheries yields (Arantes et al., 2018; Arantes, Winemiller, Asher, et al., 2019; Castello et al., 2017; Pereira et al., 2023). However, fishes respond differently to a gradient of forest loss in the Amazon River floodplain, with the magnitude and direction of response depending on functional traits (Arantes et al., 2018; Arantes, Winemiller, Asher, et al., 2019).

In Amazonian floodplains, fish rely on lake systems—local catchments that are hydrologically connected for 6–9 months each year (sensu Arantes et al., 2018; Castello et al., 2017). These systems are loosely separated by major secondary channels or natural levees and include mosaics of oxbow lakes, side channels, flooded wetlands, and depressions (Fernandes et al., 2009; Goulding, 1980; Junk et al., 1989). In floodplain lake systems, stands of floating macrophytes, or floating meadows, create fish habitat during the seasonal flood pulse (Junk, 1970). These dynamic habitats expand and contract annually with the flood pulse and cover large portions of the floodplain during peak high water (Cajado et al., 2023; Gomes et al., 2012; Melack & Forsberg, 2001; Petry et al., 2003). Floating meadows in the Amazon provide food and refuge from predators in their dense submerged root and stem networks, acting as nurseries for early life stages and resources for adults of numerous small fish species (Crampton & Hopkins, 2005; Oliveira et al., 2024; Sánchez‐Botero et al., 2001). Floating meadows in floodplain lakes are integral for the recruitment of most species of commercial fish and vital to the Amazon River fishery (Barthem & Goulding, 2007; Goulding et al., 1996; Kimura et al., 2021; Zacardi et al., 2020). Floating meadows are critical habitats for rare and threatened keystone species and species assessed by the International Union for Conservation of Nature (IUCN). These species range from near threatened to critically endangered, including the overexploited pirarucu (*Arapaima* spp. [Müller, 1843], listed as threatened as recently as 2013), ecologically vital seed‐dispersing tambaqui (*Colossoma macropomum* [Cuvier, 1816]), and important ornamental fishes, such as *Apistogramma* spp. (Regan, 1913), *Hyphessobrycon* spp. (Géry & Uj, 1987), and *Hemigrammus* spp. (Gill, 1858) (IUCN, 2024; Kirsten et al., 2012; Richard et al., 2018; Sousa et al., 2022).

Colonization and species filtering of fish assemblages in floating meadows are influenced by local, regional, and spatial factors (da Silva et al., 2024; Sánchez‐Botero et al., 2008). Temperature, dissolved oxygen, water depth, and other environmental factors are significantly associated with fish species distributions in floating meadows (Junk et al., 1983; Sánchez‐Botero et al., 2001; Tejerina‐Garro et al., 1998). Spatial factors, such as proximity to the river channel, also affect fish distributions based on dispersal tendencies (Penha et al., 2017; Virgilio et al., 2022). Additionally, the species composition and structural complexity of aquatic macrophytes influence fish assemblage structure in floating meadows (Lopes et al., 2015; Nonato et al., 2021; Virgilio et al., 2021). No one has yet examined how land cover affects fish assemblages of floating meadows in the Amazon.

We investigated relationships between forest cover and the abundance and occurrence of fish species and functional groups within floating meadows of the lower Amazon River floodplain. Associations between catches of relatively large fishes and gradients of forest cover in Amazonian floodplains have been examined (Arantes et al., 2018; Arantes, Winemiller, Asher, et al., 2019; Castello et al., 2017; Freitas et al., 2018). Analyses of fish assemblage structures along floodplain environmental gradients show complex patterns, including potential threshold responses (Baker & King, 2010; Röpke et al., 2017; Sasaki et al., 2008). No one, however, has sought evidence of threshold responses of fishes to gradients of forest cover in the Amazon River floodplain, despite such thresholds being observed in small Amazonian streams (Brejão et al., 2017; Dala‐Corte et al., 2020; Martins et al., 2021) and in floodplain ecosystems in other regions (Chen & Olden, 2020; Filgueira et al., 2016; Zhang et al., 2019). Quantifying thresholds of fish to forest cover is vital for predicting biodiversity changes in response to human impacts and supporting conservation goals (Baker & King, 2010; Brejão et al., 2017; Chen & Olden, 2020; Sasaki et al., 2015; Suding & Hobbs, 2009).

Our main objective was to test species‐level and community‐wide thresholds of change among fishes inhabiting floating meadows in relation to a gradient of 3–70% forest cover in the lower Amazon River floodplain. Specifically, we identified indicator species and quantified thresholds of fish community shifts in relation to forest cover. We first examined local fish assemblage structure in relation to forest cover and environmental factors in aquatic habitats. We analyzed these relationships according to taxonomic groupings and functional structure based on traits such as body size, life‐history strategy, and migratory strategy. We hypothesized that species and functional groups vary along a forest cover gradient, and some benefit from forested conditions, such as low water velocity, reduced wind speed, high habitat complexity, and abundant allochthonous resources (e.g., leaves, fruits, seeds, arthropods) (Junk, 1970).

If fish in floating meadows demonstrate quantifiable threshold responses to forest cover, then models could be used to predict responses to floodplain deforestation or reforestation. Several prominent biodiversity conservation plans, such as the Aichi Biodiversity Targets and the 4th RAMSAR strategic plan, include biodiversity responses to quantifiable land‐cover levels as global priorities (CBD, 2020; RAMSAR Convention Secretariat, 2016). Quantitative relationships based on reliable measures can guide effective management and foster compromises among landowners, conservationists, and other stakeholders to minimize biodiversity loss within a utilitarian land management framework (Mavrommati et al., 2016; Rondinini & Chiozza, 2010; Tear et al., 2005).

## METHODS

### Study area

**FIGURE 1:**
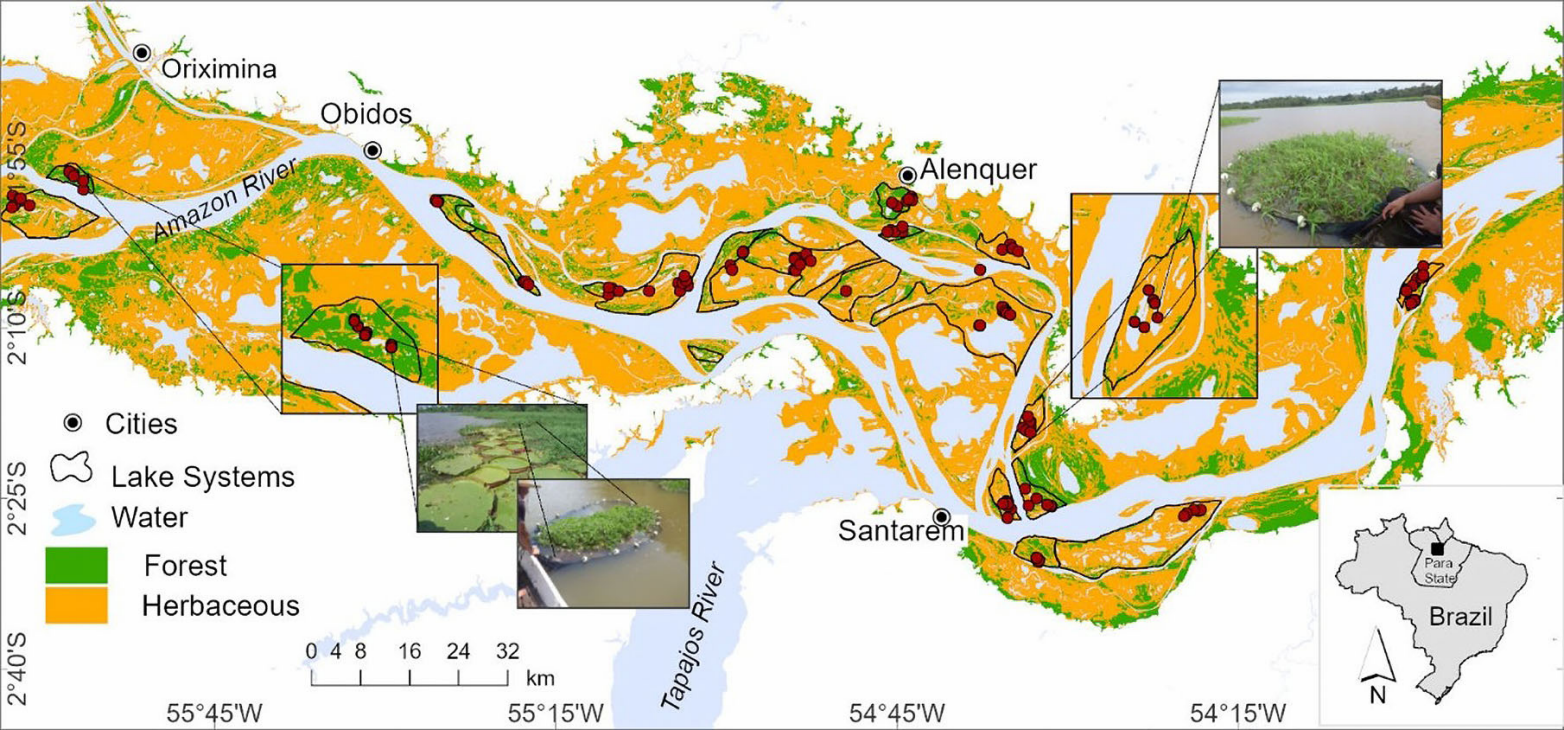
Study area in the lower Amazon River and its 19 lake systems and land cover during the low‐water period (adapted from Arantes et al. [2018]) (herbaceous, grassland cover; red dots, sampling locations; pictures, representative macrophytes and fish assemblage sampling procedure).

The study area was in the lower Amazon River floodplain in Brazil, which encompasses 17,674 ha in Pará state (Figure 1). The floodplain historically was tropical floodplain forest, but many areas were deforested centuries ago and other areas have been recently cleared and converted to grasslands that support livestock and agriculture (Nunes et al., 2019; Renó et al., 2011). During the last 30 years, 79% of the deforested area has converted to herbaceous vegetation, and 5% of the deforested area is bare soil that has not revegetated following agricultural use. The remaining 16% of the area is open water, which has increased because of bank erosion (Renó et al., 2011). Predominant trees are *Hura crepitans* (Linnaeus, 1753), *Triplaris surinamensis* (Chamisso,^1833^), *Calycophyllum spruceanum* (Bentham,^1857^), *Pseudobombax munguba* (Carvalho‐Sobrinho & Dorr, 2017), and *Ceiba pentandra* (Gaertner, 1788) (Daly & Mitchell, 2016). Dominant herbaceous vegetation includes *Heliconia* spp. (Linnaeus, 1771), *Cyclanthaceae*, *Marantaceae*, and *Zingiberaceae* (Daly & Mitchell, 2016; Junk, 1997). Aquatic habitats of the floodplain include wetlands, lakes, and secondary channels. Floating meadows in the study area were dominated by *Paspalum repens* (Bergius, 1772), *Echinochloa polystachya* (Hitchcock, 1920), *Eichhornia* spp. (Kunth,^1842^), and *Pistia stratiotes* (Linnaeus, 1753). During the annual low water period, lakes and channels retain water, and some become isolated, allowing species, such as *Pa. repens* and *Ec. polystachya*, to grow on exposed substrates (Junk, 1970, 1984). As waters rise during the annual flood pulse (magnitude averages 5.7 m above base flow for 6–9 months), macrophyte stalks break, plants drift, and some vegetation remains rooted throughout the flood period (Junk, 1997). At high water levels, lakes and secondary channels gain connections to the main river channel.

### Land cover and environmental variables

We used a remotely sensed dataset that quantified dominant land cover throughout our study area (Arantes et al., 2018; Arantes, Winemiller, Asher, et al., 2019; Hess et al., 2025) based on the following categories: forest, terrestrial herbaceous vegetation, floating meadow, and open water (Appendix S1). Hess et al. (2015) used Landsat Thematic Mapper images at 30‐m resolution (images acquired 30 November 2008 and 23 October 2009; data available in Hess et al. [2025]) to map land cover during the low‐water stage. Seven ALOS PALSAR (Advanced Land Observing Satellite‐1 Phased Array type L‐band Synthetic Aperture Radar) swaths acquired during the early rising‐water period each year from 2006 to 2010 (Hess et al., 2015) were used to map macrophyte (floating meadow) coverage. Because coverage was measured during the same season each year, within‐year seasonal variability in macrophyte coverage could not be estimated. An index quantifying the percentage of each lake system with macrophytes present in 3 or more of the 5 years analyzed was used to measure macrophyte coverage. Data from remotely sensed imagery were combined into spatial units delineated as local catchments (i.e., lake systems sensu Arantes et al. [2018] and Castello et al. [2017]) that contained various hydrologically interconnected features, such as secondary channels, wetlands, shallow lakes, grasslands, and forests. Nineteen lake systems (Figure 1) were selected that spanned a gradient of 3–70% forest cover and percentages of macrophyte, open water, and herbaceous vegetation cover. At each fish survey site, during 3 flood pulse phases in 2013–2014 (rising‐water, falling‐water, and low‐water), we measured local aquatic conditions including water depth, physicochemical water quality parameters, and macrophyte species richness (Appendix S1).

### Fish sampling

Standardized fish surveys were conducted at 369 sites in 19 lake systems along 250 km of the lower Amazon River floodplain from August 2013 to November 2014. Fish were surveyed from multiple floating meadow stands in each lake system during 3 periods of the annual flood pulse (rising water, *n* = 162; falling water, *n* = 126; dry season, *n* = 81). For each sample, a patch of floating meadow (area about 16 m^2^) was surrounded by a seine net (10 m length × 3 m depth) that was drawn to a circle in the manner of a purse seine, with the bottom lead line pulled into the boat first, to capture all fishes present. All fishes were removed and placed in plastic bins where they were euthanized and then placed in 2‐L plastic bags and labeled for preservation and storage. Specimens were later identified to species with identification keys. Some species were identified only to genus level because they were juveniles or potentially undescribed species. Applicable national and institutional guidelines for the care and use of animals were followed (approval of animal use protocol, Texas A&M University IACUC 2013‐0099 [reference number 004728] and Ministério do Meio Ambiente—MMA, Instituto Chico Mendes de Conservação da Biodiversidade—ICMBio, Sistema de Autorização e Informação em Biodiversidade [SISBIO‐Brazil] [reference number 30852–5).

### Data analyses

To analyze relationships between local fish assemblage structure and local‐scale habitat variables, we used partial redundancy analysis (pRDA) with fish abundance as a response variable. A pRDA is a constrained ordination technique used to visualize and summarize patterns in multivariate data by extracting axes (linear combinations of variables) derived from a multivariate regression model that describes how response variables are related to explanatory variables (Legendre & Legendre, 2012). Plots were created to represent relationships among fish assemblages and environmental variables in relation to the extracted axes. Environmental variables were percent open water cover, dissolved oxygen (milligrams per liter), temperature (degrees Celsius), transparency (centimeters), water depth (centimeters), percent macrophyte cover, percent forest cover, pH, conductivity (milliSiemens per meter), and macrophyte richness. We tested spatial autocorrelation between our sites by extracting axes of principal components of neighbor matrices (PCNM), a technique that uses principal coordinates analysis on a matrix of spatial relationships among sampling location coordinates to identify spatial patterns in ecological data (Borcard & Legendre, 2002). To identify which variables were significantly associated with fish abundance before inclusion in the pRDA, we used stepwise forward selection. This technique ranks variables according to their power to predict the response variable, which is based on significance level (*p* value assessed with an *F* test) and proportion of explained variance (*R* ^2^ values) (Blanchet et al., 2008). Out of 10 habitat variables, stepwise forward selection identified 9 significant predictors for fish abundance (Appendix S2), and these were selected for inclusion in the pRDA. Forward selection also identified PCNM axes 1 and 2 as having a moderate, significant effect of spatial autocorrelation on fish assemblages; therefore, we included those axes as explanatory variables in the pRDA.

Seasonality and associated changes in water level structure fish communities in Amazonian rivers (Fitzgerald et al., 2017; Silva et al., 2020); therefore, we controlled this influence on the pRDA. Rather than including season as a predictor, we controlled for its effect, allowing a direct assessment of environmental impacts on fish abundance. Because our dataset was dominated by rare species and heavily zero‐inflated, the following steps were taken to reduce noise and uncertainty in the analysis: first, rare species (occurred in <5% of sites) were removed, which left 44 species for inclusion in the pRDA (88% of species in this dataset were deemed rare) and, second, we used the Hellinger transformation on abundance data to adjust for skewed distributions and down‐weight influence of zeros while preserving compositional structure. Hellinger transformation takes the square root of the proportional differences of abundances within sites and normalizes data by constraining values between 0 and 1 (Legendre & Gallagher, 2001).

To assess potential threshold responses in individual fish and fish communities and to identify indicator species that increase or decrease with varying forest cover levels, we applied threshold indicator taxa analysis (TITAN2) (Baker & King, 2010; Baker et al., 2020). TITAN2 quantifies a species’ strength as a habitat‐specific bioindicator with indicator value scores (IndVal; Dufrêne & Legendre, 1997). These scores are a combination of the specificity to and fidelity of a species in that habitat and range from 0% (no association) to 100% (perfect indicator). These values show where a species’ abundance and occurrence rate of change is highest along an environmental gradient (i.e., the change point). It converts IndVal scores to *z* scores to include responses from low‐occurrence but highly sensitive species to account for impacts on rare taxa. In TITAN2, a bootstrap permutation test was run with 1000 iterations to provide confidence estimates for change points. Change points were interpreted as either thresholds or gradual change responses, depending on their magnitude and confidence. Narrow confidence intervals surrounding change points, high change magnitude (high *z* scores), and synchrony among change points of multiple taxa provided evidence for an assemblage change threshold (Baker & King, 2010). TITAN2 identifies both individual species‐level change points and community‐wide aggregated change points.

TITAN2 identifies indicator species based on the following criteria: species considered pure (>95% of all 1000 iterations responded with the same positive or negative direction to an increase in forest cover) and reliable (>95% of all 1000 iterations result in change points with *p* values determined at α = 0.05). Each species receives either a positive, *z*+, or negative, *z*−, designation relative to its response to an increasing forest cover gradient (*z+* species increase as forest cover increases, whereas *z*− species decrease as forest cover increases). For our purposes, *z*+ species were indicators that responded positively to increasing percentages of forest cover and negatively to decreasing percentages in forest cover. The *z*− species were indicators that responded negatively to increasing percentages of forest cover and positively to decreasing percentages of forest cover. We used TITAN2 to analyze the full dataset of 369 samples, including 146 species that occurred at least 3 times (required for TITAN2) to identify indicator species, determine their change points, and evaluate their responses to a gradient of 20 forest cover levels measured at the lake system scale. We grouped fish responses across all seasons in TITAN2 and, to check for bias, we ran separate TITAN2 analyses for each season (Appendices S7–S9).

To assess any taxonomic and functional influence on patterns of species responses to forest cover, we categorized TITAN2 response groups (*z*+ taxa or *z*− taxa) according to taxonomic family and functional groups associated with migratory strategy, life‐history strategy, and body size (standard length [SL]). Indicator taxa were divided into 3 migratory strategies based on Arantes, Winemiller, Asher, et al. (2019): sedentary (species spend their entire life in floodplain habitats [*n* = 29]), local migrators (species migrate laterally between the river and floodplain according to seasonal changes in water level [*n* = 9]), and regional migrators (species migrate longitudinally long distances [*n* = 9]). Indicator taxa were also classified by life‐history strategy based on maximum body size, size at maturation, batch fecundity, and parental care (Arantes, Winemiller, Asher, et al., 2019; Röpke et al., 2017; Winemiller & Rose, 1992). We considered 4 life‐history strategies: periodic strategists (species with high batch fecundity [>9000], no parental care, and small oocytes [*n* = 21]), opportunistic strategists (early maturation, small size, sustained reproductive effort, and no parental care [*n* = 8]), intermediate strategists (batch fecundity from 1000 to 9000, large oocytes, and intermediate parental care [*n* = 6]), and equilibrium strategists (low batch fecundity, large oocytes, and high level of parental care [*n* = 12]).

We then assessed the proportion of indicator species in each taxonomic and functional group. We also assessed the central tendency (median) change point values (percentage of forest cover at which change point occurred) for each taxonomic and functional group within each indicator group (*z*+ and *z*−). To further test functional differences in indicator fish, we compared fish body size (mm SL) between *z*+ and *z*− taxa (SL data obtained primarily from Queiroz et al. [2013]; when data were not available for this reference, we used data from FishBase [Froese & Pauly, 2000]). Visual assessment and the Shapiro test indicated that body size was not normally distributed, and a Levene test indicated that the equal variance assumption was violated between *z*+ and *z*− groups. We therefore used a Mann–Whitney *U* test to assess differences between the 2 groups. Data were analyzed and plotted in the TITAN2 package (Baker et al., 2020) and ggplot2 package R 4.1.3 (R Core Team, 2022).

## RESULTS

Stepwise forward selection revealed most of the variation in fish abundance was explained by forest cover (*R* ^2^ = 0.018) and macrophyte richness (*R* ^2^ = 0.017) (Appendix S2). The pRDA showed forest cover was strongly associated with RDA axis 1 (average score for forest in axis 1 = 0.78) and open water was strongly associated with RDA axis 2 (average score for open water in axis 2 = 0.57). Approximately 9.4% of variance in fish abundance was explained by variables other than seasonality (9 habitat variables plus 2 PCNM axes included in the pRDA) (Appendix S2), whereas seasonality independently accounted for 8.6% of variance. The complete RDA, encompassing variance adjusted for seasonality, explained cumulatively 18% of variance in fish abundance.

**FIGURE 2:**
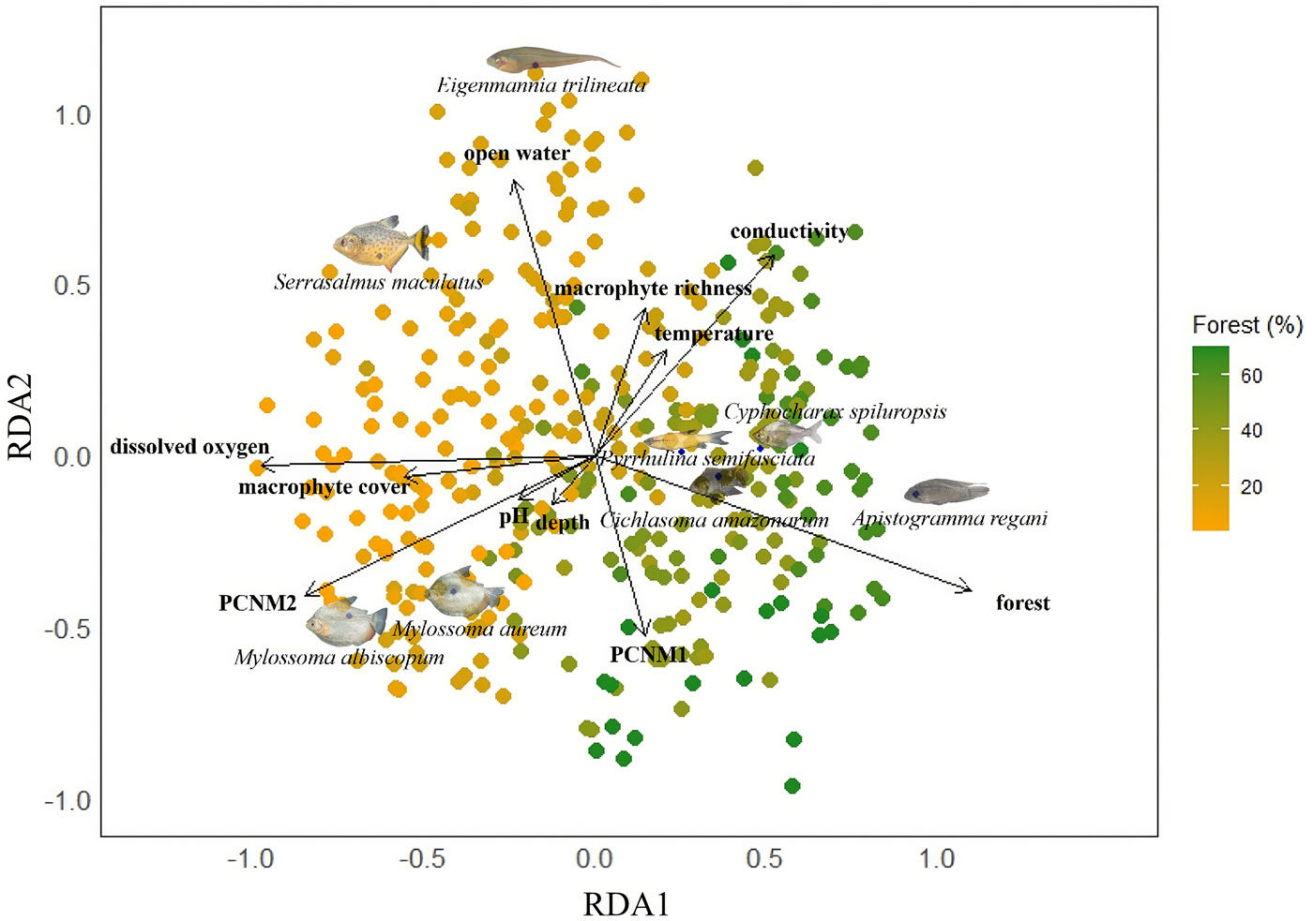
Results of partial redundancy analysis (pRDA) for RDA axes 1 and 2 showing associations between statistically significant environmental predictor variables and fish abundance as response variables (PCNM, principal coordinates of neighborhood matrix; axes, eigenvectors that capture the scale of spatial structure in our data). Species that are not at least 5% explained by environmental variables are not shown to improve readability. Scaling equal to 1 shows relationships between explanatory and response variables. Fitted site scores (colored circles) are displayed to show relationships of strictly orthogonal linear combinations of the explanatory variables (arrows).

Species most strongly associated with high levels of forest cover (Figure 2) included cichlids *Apistogramma regani* (Morales‐Betancourt, 2022) and *Cichlasoma amazonarum* (Kullander, 1983) and other families, such as Curimatidae (*Cyphocharax spiluropsis* [Eigenmann & Eigenmann, 1889]) and Triportheidae (*Triportheus angulatus* [Spix & Agassiz, 1829]). Abundance of these species was negatively associated with dissolved oxygen, macrophyte cover, and open water. Species negatively associated with forest cover included regional migrators, such as anostomid *Schizodon fasciatus* (Spix & Agassiz, 1829) and omnivorous serrasalmids *Mylossoma aureum* (Spix & Agassiz, 1829) and *Mylossoma albiscopum* (Cope, 1872). These species were positively associated with macrophyte cover and negatively associated with macrophyte richness, temperature, and conductivity. Gymnotiforms *Eigenmannia trilineata* (López & Castello, 1966), *Brachyhypopomus beebei* (Schultz, 1944), and piranha *Serrasalmus maculatus* (Kner, 1858) were negatively associated with forest cover and positively associated with open water and macrophyte richness (Figure 2).

The pRDA revealed relationships among several explanatory variables. Forest cover was negatively associated with macrophyte cover, dissolved oxygen, and open water. Macrophyte cover was negatively associated with forest cover, conductivity, macrophyte richness, and temperature, and positively associated with dissolved oxygen and water depth. Open water was positively associated with macrophyte richness and temperature and negatively associated with forest cover (Figure 2).

**FIGURE 3:**
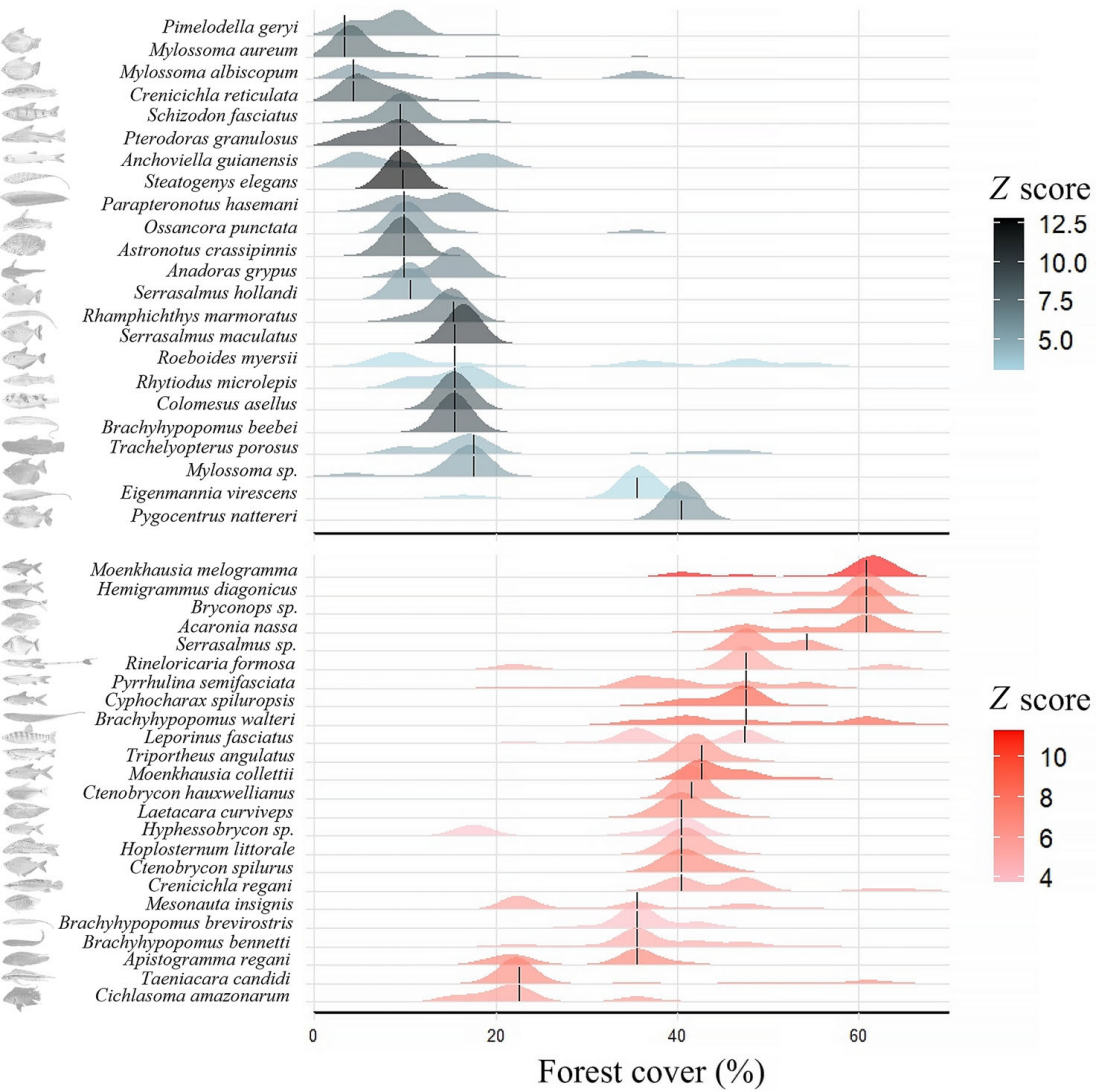
Fish species deemed pure (>95% of 1000 replicates responded in the same direction) and reliable (environmental change points are consistently significant at *p* < 0.05) indicators of forest cover over a gradient of forest cover levels through all seasons identified by threshold indicator taxa analysis (TITAN2) (red shading, species increase as levels of forest cover increase; gray shading, species decrease as levels of forest cover increase; shading gradations, the darker the shade, the greater the magnitude of species abundance and occurrence change in response to forest cover [measured in *z* scores, i.e., how distinctively fish group along the gradient]; vertical black lines, median change points [areas where species change along the gradient is maximized] for indicator taxa across replicates; height and shape of curve, density plot of bootstrapped responses for the species along the gradient; position on the *x*‐axis, where along the gradient i.e., at what percent forest cover, a significant change occurs for a species).

TITAN2 identified 47 indicator species with pure and reliable responses to the forest cover gradient (Figure 3; Appendix S3). The *z*− species (*n* = 23) tended to have narrowly defined change points at approximately 20% and 9% forest cover. The *z*− species with the highest magnitudes of change were *Steatogenys elegans* (Steindachner, 1880) (*z* = 12.8), *Se. maculatus* (*z* = 10.5), and *Pterodoras granulosus* (Valenciennes, 1821) (*z* = 10.5). The *z*+ species (*n* = 24) changed more gradually between 20% and 70% forest cover, although several species (approximately 11) responses clustered around 40% (within 7.05%) forest cover. The *z*+ species with the highest magnitudes of change were *Moenkhausia melogramma* (Eigenmann, 1908) (*z* = 11.28), *Cy. spiluropsis* (*z* = 9.50), and *Moenkhausia collettii* (Steindachner, 1882) (*z* = 9.50) (Figure 3). Running separate TITAN2 iterations for each season identified subsets of the same indicator species from the aggregated dataset (Appendices S7–S9).

**FIGURE 4:**
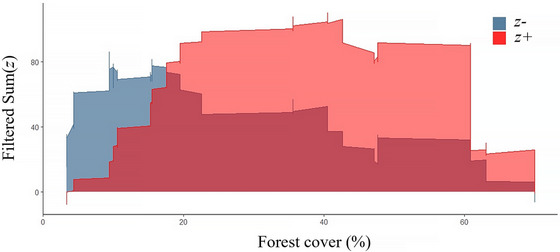
In all seasons, magnitude of change in fish community composition relative to forest cover (*z+*, species that increase as forest cover increases; *z*−, species that decrease as forest cover increases; sumz(+), summed responses of *z+* species; sumz(−), summed responses of *z*− species; peaks, areas of high change in community composition; plateaus, areas of constant change rate). Summed responses of the community are calculated using cumulative responses of each respective response group (*z*+ and *z*−) within pure (>95% of 1000 replicates responded in the same direction) and reliable (environmental change points [i.e., areas where species change along the gradient is maximized] are consistently significant at *p* < 0.05) indicator taxa.

For community threshold analysis based on the set of pure and reliable indicator species (Figure 4), TITAN2 indicated a community‐wide change point for *z*+ taxa at 40.5% forest cover (95% confidence interval [CI] 35.6–54.2). For *z*− taxa, the community change point was 9.4% forest cover (95% CI 9.4–17.5). For the entire community including taxa not deemed pure or reliable (Appendix S4), the community change point for *z*+ taxa was 40.5% forest cover (95% CI 29.0–47.6), and for *z*− taxa, the change point was at 10.2% forest cover (95% CI 9.9–15.4). There was high consistency among change points of *z*− taxa, with a narrow spread across replicates and narrow confidence intervals. The *z*+ taxa were more variable, with change points spread broadly along the gradient and several around 40% forest cover. The pivotal community change point for both *z*+ and *z*− was 20% forest cover. Increasing forest cover past this point resulted in rapidly declining *z*− taxa and gradually rising *z*+ taxa (Figure 4).

**FIGURE 5:**
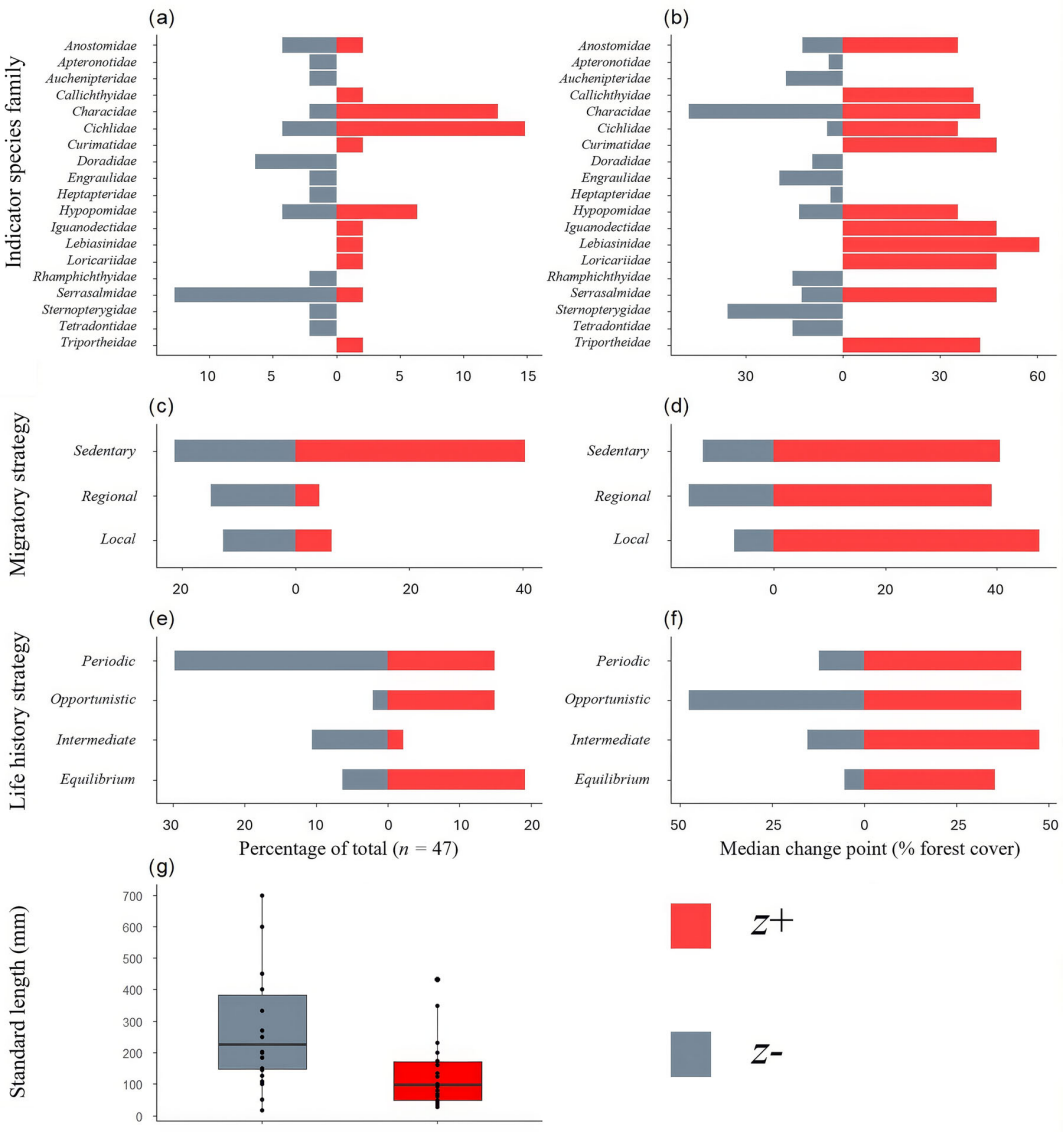
Percentage of the total number of indicator fish species (a) in taxonomic families, (c) by migratory strategy, and (e) by life‐history strategy; median in‐group change points (areas where species change along the gradient is maximized) of fishes to a gradient of forest for (b) taxonomic families, (d) migratory strategies, and (f) life‐history strategies (*z*+, species that increase as forest cover increases; *z*−, species that decrease as forest cover increases); and (g) distribution of standard lengths of indicator fishes (circles, individual data points; box extent, interquartile range; horizontal line, median; whiskers, minimum and maximum values within 1.5 times the interquartile).

The families with the highest proportions of *z*+ taxa were Characidae (85.7% *z+*) and Cichlidae (77.8% *z+*). The families with the highest proportions of *z*− taxa were Doradidae (100%) and Serrasalmidae (85.7%). Most regional migrators (77.8%) were *z*−, whereas only 22.2% of regional migrators were *z+* taxa. Most local migrators were *z*− (66.7%), and 33.33% were *z*+ taxa. Most sedentary species were *z*+ (62%), and 38% of sedentary species were *z*−. Most periodic strategists were *z*− taxa (66.7%), and 33.3% were *z*+. Similarly, 83.3% of intermediate strategists were *z*− and 16.7% were *z*+. Opportunistic strategists were mostly *z*+ (87.5%), and 12.5% were *z*−. Most equilibrium strategists were *z*+ (75.0%), and 25.0% were *z*−. In all functional and taxonomic groupings, *z*+ taxa had higher median change points than *z*− taxa relative to forest cover species (Figure 5b,d,f; Appendix S3). Average body length of *z*− species (204 mm [SE 124], *n* = 23) was greater than the average of *z+* species (128 mm [SE 118], *n* = 24) (Figure 5g; Appendix S3), a difference that was statistically significant (*U* test *p* < 0.01), with medium effect size (*r* = 0.37).

## DISCUSSION

We identified thresholds and zones of gradual change of indicator species inhabiting floating meadows to a gradient of forest cover in the lower Amazon River. Due to wide confidence intervals and inconsistent change points among indicator fish species that increased with forest cover, we concluded that 40% forest cover represents a gradual transition in fish assemblage structure rather than a discrete threshold. Our results indicated that below this level, further reductions in forest cover may result in declines in the abundance and occurrence of several functional and taxonomic groups. In contrast, 9% and 20% forest cover were thresholds at which fish assemblage structure shifted sharply, marked by significant changes with narrow confidence intervals. Below 20% cover, several functional and taxonomic groups of fish assemblages in floating meadows increased rapidly in occurrence and abundance. Overall, observed species‐specific and community‐wide thresholds in relation to forest cover were consistent with the view that floodplain forest cover influences the structure of fish assemblages through environmental filtering in the Amazon (Arantes, Winemiller, Asher, et al., 2019; Freitas et al., 2018; Montag et al., 2019).

We suspect forest cover affects fish in floating meadows primarily by altering nutrient dynamics and aquatic macrophyte growth, which influence structural complexity and food availability. Forest cover was negatively associated with macrophyte cover, suggesting areas with lower forest cover have greater compensatory growth of floating meadows due to greater light availability, as documented by Fares et al. (2020). In addition to receiving more sunlight that supports autochthonous production, areas with low forest cover probably receive lower allochthonous inputs. In forested Amazonian floodplain lakes, local allochthonous inputs are high, comprising most of the suspended organic matter and nutrient inputs (Ertel et al., 1986; Hedges et al., 1986; Sobrinho et al., 2016). Although fruits, seeds, flowers, and terrestrial animals that fall in the water provide food for adult fish, forest canopy shade and low dissolved inorganic nutrients often limit autochthonous basal production, reducing food resources for juvenile fish (Bayley, 1989). Floating meadows in lakes with high forest cover accumulate decomposing allochthonous material in their submerged roots and stems, forming a soil‐like detrital substrate (Junk, 1970). Although this material is eaten by shrimp and other aquatic invertebrates, which in turn feed fish, it supports less fish biomass than microalgae attached to surfaces or suspended in the water column in sunlit waters (Bayley, 1989). The movement of material, nutrients, and energy connecting aquatic and terrestrial habitats can have significant effects on food web dynamics (Nakano & Murakami, 2001).

Abiotic conditions, such as current and wind velocities, may further differentiate ecosystem properties of floating meadows in forested and nonforested areas (Carr et al., 1997; Thomaz et al., 2003). These abiotic conditions probably regulate, to some extent, physical characteristics of floating meadows, including structural complexity. Structural complexity in Amazonian floating meadows acts as an environmental filter, favoring species with highly maneuverable swimming abilities (Nonato et al., 2021). We therefore infer that the relative level of forest cover affects macrophyte growth, nutrient availability, and structural complexity in floating meadows, which in turn influence the patterns we found in fish assemblages.

### Fish species associations with local environmental variables

Environmental filtering during local community assembly was supported by results from our pRDA analysis. This analysis identified species strongly associated with greater forest cover (40–70% forest cover)—in particular, 2 cichlids (*Ap. regani* and *Ci. amazonarum*) and characid *Cy. spiluropsis*. These fishes are often found in floating meadows in undisturbed floodplain lakes (Zuanon et al., 2004), and some exploit allochthonous food resources (Martins et al., 2021; Zuanon et al., 2015). This observation is consistent with results from our threshold analyses, which showed these species were indicators of increasing forest cover. Conversely, several gymnotiforms (*Eig. trilineata*, *B. beebei*) and piranhas (e.g., *Se. maculatus*) were negatively associated with forest cover (most abundant at sites <20% forest cover) and positively associated with open water. *Eigenmannia* spp., unlike most floodplain‐dwelling gymnotiforms, often leave structurally complex daytime refugia to forage at night on zooplankton in the water column (Crampton, 1998; Winemiller & Adite, 1997) and thus may be less reliant on structural complexity offered by floating meadows in forested environments. Piranhas, such as *Se. maculatus*, identified as indicators of decreasing forest cover, exploit diverse food resources from a variety of aquatic habitats (Correa et al., 2015; Goulding, 1980) and are therefore more tolerant of forest loss. Likewise, *Sc. fasciatus*, *My. aureum*, and *My. albiscopum* were negatively associated with forest cover (most abundant at sites <20% forest cover); they had positive associations with total floating meadow cover. Juveniles of these species use floating meadows as nurseries (Sánchez‐Botero et al., 2001), and adults directly ingest aquatic macrophytes (Arantes, Winemiller, Petrere, et al., 2019; Zuanon & Ferreira, 2008). It is possible these species found higher quality nursery habitat in less‐forested floating meadows.

### Indicator species taxonomic and functional group responses

Our principal objective was to test for threshold responses among fish communities in floating meadows to a gradient of forest cover. We identified change points for 47 indicator species (out of 278 species in the full survey dataset). However, only those with narrow confidence intervals and high *z* scores were interpreted as threshold responses. Species with wider confidence intervals and lower *z* scores exhibited more gradual changes in frequency and abundance along the forest cover gradient. Both gradual change points and abrupt threshold responses could be useful for inferring how fishes respond to deforestation in the Amazon floodplain. Indicator species displayed functional and taxonomic group response patterns, consistent with prior studies showing divergent fish responses to forest cover gradients in Amazonian rivers, floodplains, and streams (Arantes, Winemiller, Asher, et al., 2019; Brejão et al., 2017; Ilha et al., 2019; Lugo‐Carvajal et al., 2023).

In our study, the abundance of several species of families Characidae and Cichlidae was positively associated with forest cover. The *z*+ characids were small omnivores (e.g., *Ctenobrycon* [Eigenmann, 1908] and *Moenkhausia* [Eigenmann, 1903] spp.) common in floating meadows surrounded by forested floodplains (Winemiller & Jepsen, 1998; Zuanon et al., 2004). Henderson and Hamilton (1995) found certain species of dwarf cichlids (*Apistogramma* spp.) to be strongly associated with floating meadows, and this genus was previously identified as an indicator of forested habitat (Martins et al., 2021). In forest streams, dwarf cichlids typically inhabit structurally complex microhabitats, such as submerged leaf packs, branches, and root wads (Zuanon et al., 2015). In our study, most cichlids were adversely affected by forest loss, presumably due to reliance on allochthonous inputs. However, the cichlids *Astronotus crassipinnis* (Heckel, 1840) and *Crenicichla reticulata* (Heckel, 1840) were positively associated with low forest cover. These comparatively large predators capture small fishes, likely requiring different habitat than smaller cichlids to hunt. The only characid that decreased as forest cover increased was the scale feeder *Roeboides myersii* (Gill, 1870); it likely has different habitat requirements than other characids because of its feeding niche.

Functional groups revealed more consistent associations with forest cover than did fish taxonomic groupings. Species that were relatively small and sedentary, with equilibrium or opportunistic life‐history strategies, tended to have lower occurrence and abundance in areas with low forest cover. This pattern was observed among several cichlid species, likely owing to their equilibrium life‐history strategies that involve nesting and brood guarding near submerged structures (Arantes, Winemiller, Asher, et al., 2019; Carvalho De Lima & Araujo‐Lima, 2004). Similarly, certain opportunistic strategists, including many characids, were less frequent and abundant in floating meadows where there was low forest cover. These species likely rely on allochthonous food resources (Leal et al., 2018) or use the structural complexity of the flooded forests adjacent to the floating meadows. Conversely, species reliant on forest cover may be poorly adapted to habitats with intense sunlight and extensive, homogenous aquatic vegetation, instead thriving in or near edge habitats with small patches of floating meadows in forested areas.

Migratory and relatively large species with periodic and intermediate life‐history strategies tended to have higher occurrence frequencies and abundances in areas with lower forest cover. Many periodic strategists are large, are relatively long‐lived, and have high batch fecundity with seasonally restricted spawning periods (Winemiller & Rose, 1992). Although some periodic strategists identified as *z*− species enter flooded forests as adults to feed, their juveniles use floating meadows as nursery habitat. The prevalence of juveniles of these groups in low forest cover areas contrasts with earlier studies showing their adult stages were positively linked to forest cover in the lower Amazon (Arantes, Winemiller, Asher, et al., 2019). Those studies predominantly captured large‐bodied fishes in gillnets, whereas our seine surveys captured mostly small fishes, including juveniles of large species. Based on findings from these studies, it seems apparent that juveniles and adults of periodic and migratory species respond to forest cover differently in the lower Amazon.

Functional groups generally responded consistently to forest cover, but some groups, such as periodic strategists, showed exceptions due to ecological diversity. Although most periodic strategists in our study were large‐bodied species and associated with decreasing forest cover, about one‐third were *z*+ indicators. These were mainly small‐bodied species, such as *Brachyhypopomus* spp. (Mago‐Leccia, 1994), *Bryconops* spp. (Kner, 1858), and *Cy. spiluropsis*, that are associated with floating meadows during all life stages (Lima et al., 2003; Sánchez‐Botero et al., 2001; Zuanon & Ferreira, 2008; Zuanon et al., 2004).

### Limitations and future research

We found that small, sedentary, equilibrium, and opportunistic life‐history strategist fishes were mainly indicators of increasing forest cover, whereas large, migratory, periodic, and intermediate life‐history strategist fishes—primarily juveniles in our samples—tended to be indicators of decreasing forest cover. In contrast, prior research shows that low forest cover in the Amazon floodplain is generally associated with low abundance of large migratory, periodic strategist fishes (Arantes et al., 2018; Arantes, Winemiller, Asher, et al., 2019; Castello et al., 2017; Freitas et al., 2018; Pereira et al., 2023). This suggests that, even if low forest cover were to favor juveniles of these fishes in floating meadows, adult survival and growth are probably adversely affected. Future research should examine micro‐ to macrohabitat requirements of all life stages of large, migratory, periodic strategists in the Amazon River. Also of interest is the ecological functions of the structural complexity of floating meadows compared with that of submerged forest structures (roots, branches, leaves) and the importance of autochthonous and allochthonous resources in aquatic food webs. Another area requiring research is the relationship between fish recruitment and the characteristics of floating meadows and their surrounding landscapes (Cheminée et al., 2015; Hinz et al., 2023).

Our study lacked sites with relatively pristine land cover (70–100% forest). Some fishes respond to low levels of forest loss (Brejão et al., 2017; Dala‐Corte et al., 2020; Martins et al., 2021). Locations with >70% forest cover probably have fish communities even more sensitive to reductions in forest cover than those we observed. Deforestation in the Amazon floodplain has been pervasive (Nunes et al., 2019), and our dataset reflects areas with significant, long‐term forest loss, which leads to fish communities dominated by species relatively tolerant of reduced forest cover. Some species undoubtedly benefit from sparse forest cover in the Amazon floodplain. The negative association between forest and floating meadow cover suggests that aquatic macrophyte growth and aquatic primary productivity are enhanced by forest loss (Silva et al., 2010). Although we inferred the impacts of forest loss on fish communities, we did not directly assess effects of deforestation. To assess how deforestation alters community structure, future studies could sample assemblage composition of floating meadows before and after deforestation events.

Seasonality is a major driver of fish assemblage structure in the Amazon (Fitzgerald et al., 2017; Silva et al., 2020). To test seasonality bias, we conducted separate TITAN2 analyses by season, which identified indicator species and responses that were subsets of the aggregated dataset (Appendices S7–S9). This consistency suggests that aggregating seasonal data in TITAN2 did not bias our assessment; rather, it captured year‐round responses of fish. For our analysis, floating meadow cover was measured during one season (early rising water), whereas fish sampling occurred across 3 seasons within a given year. Floating meadow cover fluctuates with the seasonal flood pulse (Junk, 1970), and our floating meadow estimates should have approximated their maximum annual extent. We averaged floating meadow coverage across 3 years of remotely sensed imagery to provide a reliable metric to estimate variation across lake systems.

### Conservation implications

In increasingly human‐altered environments, conservation strategies based on pristine ecosystems are often impractical. Instead, integrating natural systems with human use offers more sustainable long‐term solutions (Mavrommati et al., 2016). Land‐cover thresholds provide actionable guidelines for sustainable human land use while enabling direct analysis of ecological changes (Suding & Hobbs, 2009). These ecological thresholds are particularly valuable for conservation because they align ecological goals with policy and economic frameworks, ensuring a balance between preservation and development.

Our results revealed forest cover thresholds and critical zones of transition that influence fish communities in floating meadows. Even if thresholds are not identified, using TITAN2 to identify gradual zones of decline is valuable for land management within an adaptive management framework. For example, 40% forest cover was estimated to be a critical zone below which there is a steep reduction in the abundance of multiple species. Additionally, assemblage‐wide thresholds for indicator fishes predicted rapid assemblage transition at approximately 20% and 9% forest cover. Using these thresholds, cost–benefit analyses of forested floodplains may show that certain reforestation efforts might not be effective in restoring native fish assemblages until a certain threshold of forest cover has been attained, allowing for more efficient use of resources and time.

Monitoring indicator species in addition to water quality parameters provides a more comprehensive means for assessing ecosystem status (Beck & Hatch, 2009; Grinstead et al., 2022). Biotic indicators facilitate rapid evaluation of emerging impacts on threatened species and ecosystem processes and services (Karr et al., 1986; Mundahl & Simon, 1998). Based on our results, individual species, including *Mo. melogramma*, *Hemigrammus diagonicus* (Mendonça & Wosiacki, 2011), and *Acaronia nassa* (Heckel, 1840), may be more sensitive to forest loss, whereas other species, such as *Se. maculatus* and *Pt. granulosus*, may be more tolerant. These indicator species can serve as sentinels of environmental degradation, informing policies for protection of native fish diversity, including economically and culturally important stocks. Functional groups we identified could serve as robust indicators of ecological response to changes in forest cover across regions in the Amazon floodplain (Arantes et al., 2018; Arantes, Winemiller, Asher, et al., 2019; Van den Brink et al., 2011). For example, the loss of sedentary, equilibrium strategists and smaller‐bodied cichlids would be an early warning of a major fish assemblage transition in response to initially low levels of deforestation, and an increase in juvenile species of large‐bodied periodic and intermediate strategists and migratory fishes could be indicative of late‐stage ecosystem state change.

In the lower Amazon floodplain, fish assemblages in floating meadows (this study) and aquatic habitats generally (Arantes et al., 2018; Arantes, Winemiller, Asher, et al., 2019) are significantly associated with forest cover, making consideration of this relationship essential for conservation planning (Michanek et al. et al., 2018; Putz et al., 2001). Large areas of the lower Amazon floodplain have been deforested, and, although agriculture will likely continue in fertile floodplains, there may be opportunities to establish riparian or floodplain buffer zones, target key areas for reforestation, and establish monitoring programs in support of conservation goals (Ewers et al., 2024; Suding & Hobbs, 2009). Forest protection and restoration efforts should strategically target areas approaching key forest cover thresholds for biological indicators, prioritizing sites with vulnerable species, such as those listed on the IUCN Red List (Ewers et al., 2024; Guénette & Villard, 2005). Modeling thresholds for ecological transitions in response to environmental gradients, such as land cover, and long‐term monitoring of indicator taxa and functional groups have great potential to enhance conservation in Amazonia and globally.

## Supporting information



Supplementary Material

Supporting Information

Supporting Information

Supporting Information

Supporting Information

Supporting Information

Supporting Information

Supporting Information

Supporting Information

Supporting Information
