# Supplementary material for: Threshold responses of floating meadow fish communities to floodplain forest cover in the lower Amazon River
Source: Conserv Biol. 2025 Aug 15;39(6):e70110. doi: 10.1111/cobi.70110 (PMC12658946; doi:10.1111/cobi.70110)
Supplement: Supplementary file 1 — Supplementary Material [file COBI-39-e70110-s010.docx]

**Supplementary Materials**

**Supplementary Tables**

Appendix S1. Environmental predictor variables used in our analyses, their descriptions, median values, upper quartile values, and lower quartile values.

| **Variable** | **Description** | **Median Value** | **LQ** | **UQ** |
| --- | --- | --- | --- | --- |
| Land-cover Variables |  |  |  |  |
| Forest Cover % | Percentage of tree, shrub, or semi-shrub cover that is closed-­canopy tree cover in the lake system | 22.53 | 10.51 | 47.10 |
| Herbaceous Vegetation % | Percentage of grasses, forbs, fresh sediments, or soil during the dry season in the lake system | 59.55 | 48.20 | 74.54 |
| Floating Meadow % | Percentage of the lake system with macrophytes, calculated using indices of the percent of macrophytes present (during late December to January) in three or more of 5 years analyzed (2006/2007 to 2010/2011) | 13.02 | 18.88 | 24.01 |
| Open Water % | Percentage of open water in lake systems during the dry season in (excluding the mainstem Amazon channel) | 9.28 | 3.03 | 12.86 |
| Local Environmental Variables | | |  |  |
| Macrophyte Richness | Number of macrophyte species in the sampled floating meadow | 2 | 1 | 3 |
| pH | pH where the floating meadow sample was taken | 7.10 | 6.53 | 7.60 |
| Depth (cm) | Depth in centimeters where the floating meadow sample was taken | 210.00 | 130.00 | 290.00 |
| Conductivity (mS/m) | Conductivity, in millisiemens/meter where the floating meadow sample was taken | 61.07 | 48.40 | 73.33 |
| Dissolved Oxygen (mg/L) | Dissolved oxygen in milligrams/Liter where the floating meadow sample was taken | 3.01 | 1.63 | 4.33 |
| Temperature (°C) | Temperature in Celsius where the floating meadow sample was taken | 29.67 | 28.93 | 30.57 |
|  |  |  |  |  |
|  |  |  |  |  |
|  |  |  |  |  |
|  |  |  |  |  |

Appendix S2. Continuous environmental predictor variables for fish abundance chosen by stepwise forward selection for the pRDA.

| **Variable** | **R^2^** | **R^2^ Cumulative** | **Adjusted R^2^ Cumulative** | **F Statistic** | **p Value** |
| --- | --- | --- | --- | --- | --- |
| Macrophyte Richness | 0.017 | 0. 017 | 0.014 | 6.288 | > 0.001 |
| Forest Gradient | 0.018 | 0.035 | 0.029 | 6.612 | > 0.001 |
| pH | 0.010 | 0.045 | 0.037 | 3.867 | > 0.001 |
| Depth | 0.009 | 0.054 | 0.043 | 3.495 | > 0.001 |
| PCNM Axis 2 | 0.008 | 0.062 | 0.049 | 3.215 | > 0.001 |
| Conductivity | 0.008 | 0.071 | 0.055 | 3.240 | > 0.001 |
| PCNM Axis 1 | 0.008 | 0.079 | 0.061 | 3.155 | > 0.001 |
| Open Water | 0.007 | 0.086 | 0.066 | 2.842 | > 0.001 |
| Dissolved Oxygen | 0.007 | 0.093 | 0.070 | 2.648 | > 0.001 |
| Temperature | 0.005 | 0.098 | 0.073 | 2.147 | > 0.001 |
| Floating Meadow Cover | 0.005 | 0.103 | 0.076 | 2.029 | > 0.001 |

Appendix S3. Indicator species identified by TITAN2 analysis, relative TITAN2 groupings (‘*z*+’ = indicator species that increase with increasing forest cover and ‘*z*-‘ = indicator species that decrease with increasing forest cover), environmental change point (percent forest cover), standardized z-score, family, standard length, migratory strategy, and life history strategy.

| **Species** | **TITAN2 Group** | **Change point (% Forest)** | **Z-score** | **Family** | **Standard Length (mm)** | **Migratory Strategy** | **Life History Strategy** |
| --- | --- | --- | --- | --- | --- | --- | --- |
| *Acaronia nassa* | *z+* | 60.850 | 8.10 | Cichlidae | 172 | Sedentary | Equilibrium |
| *Anadoras grypus* | *z-* | 9.420 | 6.65 | Doradidae | 146 | Sedentary | Equilibrium |
| *Anchoviella guianensis* | *z-* | 19.490 | 5.26 | Engraulidae | 52 | Regional | Periodic |
| *Apistogramma regani* | *z+* | 35.560 | 7.51 | Cichlidae | 49 | Sedentary | Equilibrium |
| *Astronotus crassipinnis* | *z-* | 9.420 | 8.57 | Cichlidae | 200 | Sedentary | Equilibrium |
| *Brachyhypopomus beebei* | *z-* | 17.550 | 9.09 | Hypopomidae | 250 | Sedentary | Periodic |
| *Brachyhypopomus bennetti* | *z+* | 35.560 | 5.36 | Hypopomidae | 232 | Sedentary | Periodic |
| *Brachyhypopomus brevirostris* | *z+* | 35.560 | 4.16 | Hypopomidae | 432 | Sedentary | Periodic |
| *Brachyhypopomus walteri* | *z+* | 60.850 | 9.36 | Hypopomidae | 175 | Sedentary | Periodic |
| *Bryconops sp.* | *z+* | 47.610 | 8.01 | Iguanodectidae | -- | Local | Periodic |
| *Cichlasoma amazonarum* | *z+* | 35.560 | 6.30 | Cichlidae | 135 | Sedentary | Equilibrium |
| *Colomesus asellus* | *z-* | 15.440 | 8.67 | Tetradontidae | 128 | Sedentary | Periodic |
| *Crenicichla regani* | *z+* | 35.560 | 5.79 | Cichlidae | 79 | Sedentary | Equilibrium |
| *Crenicichla reticulata* | *z-* | 4.350 | 8.56 | Cichlidae | 250 | Sedentary | Equilibrium |
| *Ctenobrycon hauxwellianus* | *z+* | 42.610 | 6.96 | Characidae | 70 | Sedentary | Opportunistic |
| *Ctenobrycon spilurus* | *z+* | 40.460 | 7.44 | Characidae | 63 | Sedentary | Opportunistic |
| *Cyphocharax spiluropsis* | *z+* | 47.610 | 9.50 | Curimatidae | 97 | Local | Periodic |
| *Eigenmannia virescens* | *z-* | 35.560 | 3.08 | Sternopygidae | 450 | Sedentary | Periodic |
| *Hemigrammus diagonicus* | *z+* | 60.850 | 6.90 | Characidae | 28 | Sedentary | Opportunistic |
| *Hoplosternum littorale* | *z+* | 40.460 | 5.96 | Callichthyidae | 160 | Sedentary | Equilibrium |
| *Hyphessobrycon sp.* | *z+* | 40.460 | 3.75 | Characidae | -- | Sedentary | Opportunistic |
| *Laetacara curviceps* | *z+* | 35.560 | 7.09 | Cichlidae | 100 | Sedentary | Equilibrium |
| *Leporinus fasciatus* | *z+* | 35.560 | 4.42 | Anostomidae | 350 | Regional | Periodic |
| *Mesonauta insignis* | *z+* | 22.530 | 5.88 | Cichlidae | 124 | Sedentary | Equilibrium |
| *Moenkhausia collettii* | *z+* | 42.610 | 9.50 | Characidae | 47 | Sedentary | Opportunistic |
| *Moenkhausia melogramma* | *z+* | 60.850 | 11.28 | Characidae | 40 | Sedentary | Opportunistic |
| *Mylossoma albiscopum* | *z-* | 3.330 | 6.13 | Serrasalmidae | 250 | Regional | Periodic |
| *Mylossoma aureum* | *z-* | 3.330 | 7.95 | Serrasalmidae | 200 | Regional | Periodic |
| *Mylossoma sp.* | *z-* | 17.550 | 5.50 | Serrasalmidae | -- | Regional | Periodic |
| *Ossancora punctata* | *z-* | 9.940 | 6.07 | Doradidae | 102 | Sedentary | Intermediate |
| *Parapteronotus hasemani* | *z-* | 4.350 | 6.70 | Apteronotidae | 400 | Local | Periodic |
| *Pimelodella geryi* | *z-* | 3.840 | 6.80 | Heptapteridae | 108 | Local | Periodic |
| *Pterodoras granulosus* | *z-* | 4.350 | 10.50 | Doradidae | 700 | Local | Periodic |
| *Pygocentrus nattereri* | *z-* | 40.460 | 6.06 | Serrasalmidae | 333 | Regional | Intermediate |
| *Pyrrhulina semifasciata* | *z+* | 60.850 | 6.81 | Lebiasinidae | 40 | Sedentary | Opportunistic |
| *Rhamphichthys marmoratus* | *z-* | 15.440 | 7.71 | Rhamphichthyidae | 600 | Local | Periodic |
| *Rhytiodus microlepis* | *z-* | 15.280 | 3.69 | Anostomidae | 400 | Regional | Periodic |
| *Rineloricaria formosa* | *z+* | 47.610 | 5.70 | Loricariidae | 93 | Sedentary | Equilibrium |
| *Roeboides myersii* | *z-* | 47.610 | 3.02 | Characidae | 18 | Sedentary | Opportunistic |
| *Schizodon fasciatus* | *z-* | 9.420 | 7.43 | Anostomidae | 400 | Regional | Periodic |
| *Serrasalmus hollandi* | *z-* | 9.940 | 5.48 | Serrasalmidae | 185 | Local | Intermediate |
| *Serrasalmus maculatus* | *z-* | 15.440 | 10.50 | Serrasalmidae | 202 | Local | Intermediate |
| *Serrasalmus sp.* | *z+* | 47.610 | 7.25 | Serrasalmidae | -- | Local | Intermediate |
| *Steatogenys elegans* | *z-* | 9.420 | 12.82 | Hypopomidae | 270 | Sedentary | Periodic |
| *Taeniacara candidi* | *z+* | 22.530 | 7.18 | Cichlidae | 33 | Sedentary | Equilibrium |
| *Trachelyopterus porosus* | *z-* | 17.550 | 4.75 | Auchenipteridae | 150 | Sedentary | Intermediate |
| *Triportheus angulatus* | *z+* | 42.610 | 6.64 | Triportheidae | 200 | Regional | Periodic |
|  |  |  |  |  |  |  |  |

**Supplementary Figures**


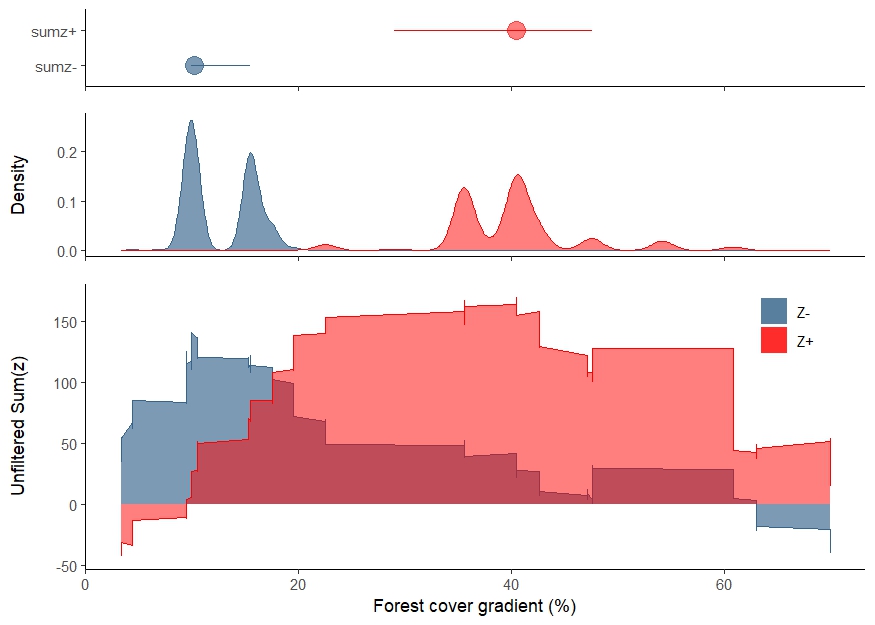


Appendix S4. Entire species community responses, including species not deemed indicator species throughout all seasons. Dark values (z-) indicate species that increase as forest cover decreases. Red values (z+) indicate species that decrease as forest cover decreases. Summed responses of the community are calculated using cumulative responses of each respective response group. In this figure, summed responses of taxa that increase with forest cover are sumz(+), and summed responses of taxa that decrease with forest cover are sumz(-). Bottom panel shows magnitude of change along the forest cover gradient in taxa that increase and decrease in the community. Peaks along the x axis indicate areas of a high change in community composition along the forest gradient. Plateaus denote regions of similar change. Middle panel is the estimated probability sum(z) across all replicates (revealing highest change points for the entire community). A narrow spread indicates high degree of precision in locating the change points. Top panel shows the observed sum(z+) and sum(z-) maxima points and their 95% CI.


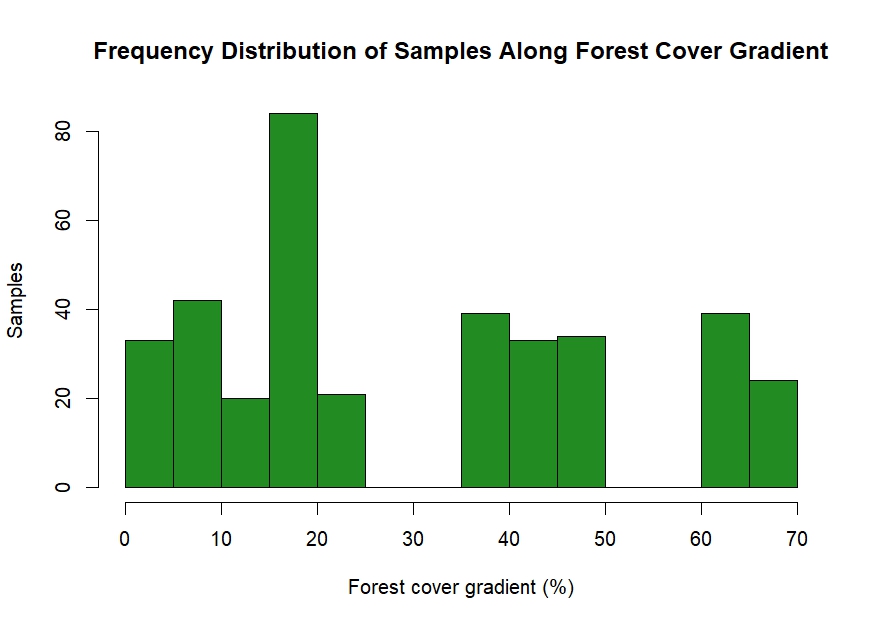


Appendix S5. Frequency distribution of samples along the gradient of forest cover percentage in the dataset used for TITAN2.


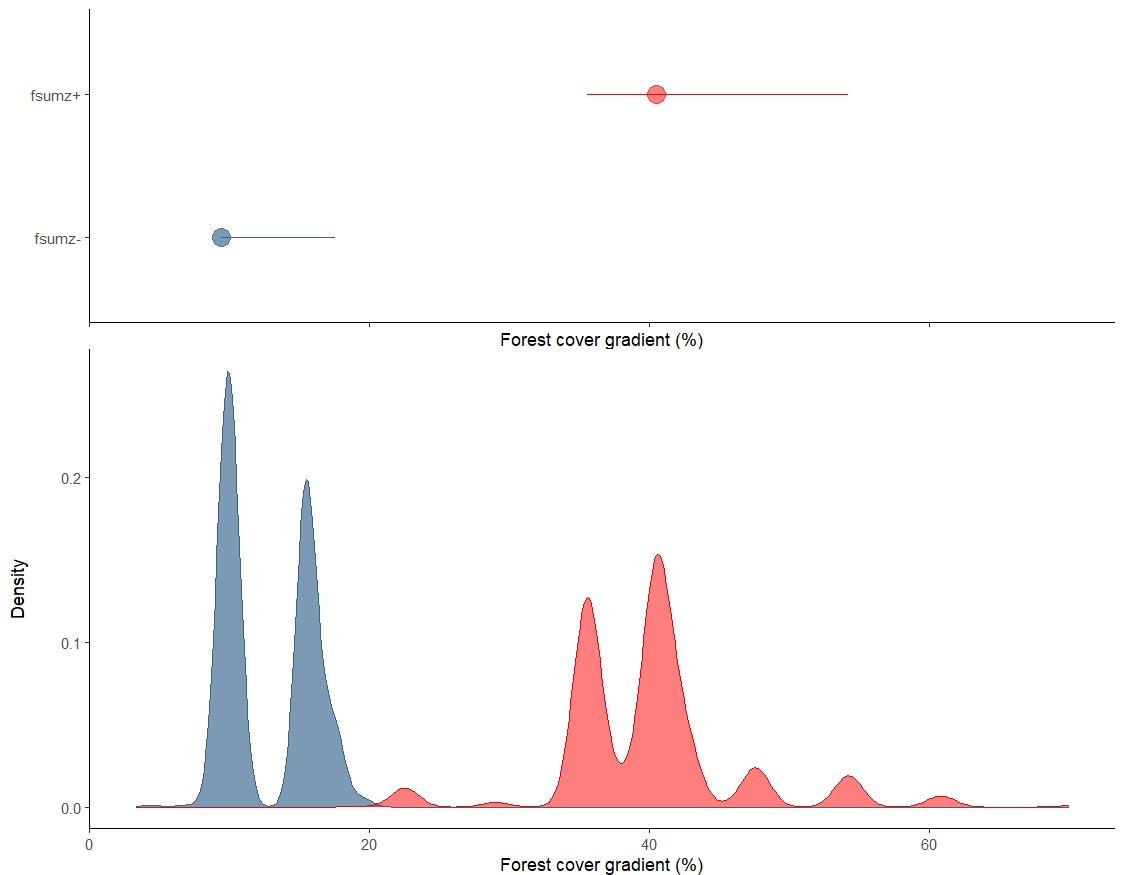


Appendix S6. Indicator species community responses throughout all seasons. Dark values (z-) indicate species that increase as forest cover decreases. Red values (z+) indicate species that decrease as forest cover decreases. Summed responses of the community are calculated using cumulative responses of each respective response group. Bottom panel is the estimated probability sum(z) across all replicates (revealing highest change points for the entire community). A narrow spread indicates high degree of precision in locating the change points. Top panel shows the observed sum(z+) and sum(z-) maxima points and their 95% CI.


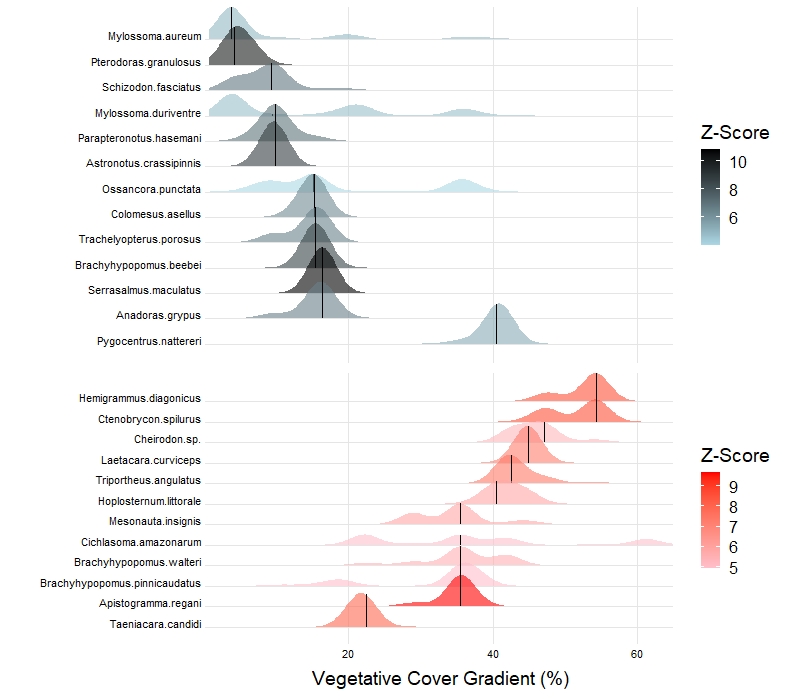


Appendix S7. Pure (> 95% of all 1000 replicates responded the in the same + or – direction to an increase in forest cover) and reliable (bootstrapped environmental change points (CPs) IndVal scores consistently result in p < 0.05) indicator species for a gradient of forest cover identified by TITAN2 throughout the rising water season. Dark values indicate species that decrease with increasing levels of forest cover. Red values indicate species that increase with increasing levels of forest cover. Z-score (color darkness) indicates magnitude of change point along the x axis (how distinctively fish are grouping along the gradient—or magnitude of change). The X axis positioning indicates where along the gradient (at what % forest cover) a significant change point occurs for those specific taxa. Vertical black lines represent median change points for indicator taxa across replicates. The shape of data represents the acceleration of change in Z-scores.


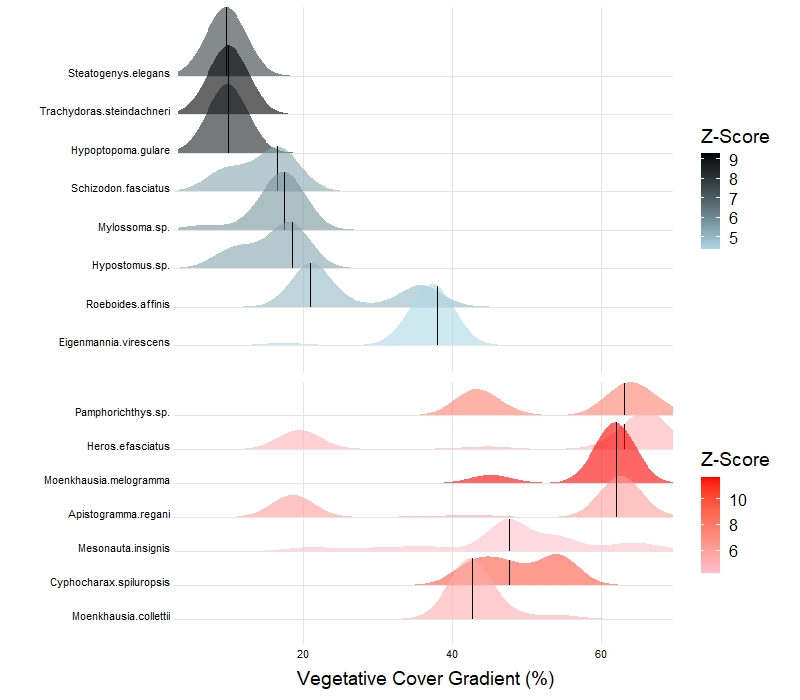
Appendix S8. Pure (> 95% of all 1000 replicates responded the in the same + or – direction to an increase in forest cover) and reliable (bootstrapped environmental change points (CPs) IndVal scores consistently result in p < 0.05) indicator species for a gradient of forest cover identified by TITAN2 throughout the dry season. Dark values indicate species that decrease with increasing levels of forest cover. Red values indicate species that increase with increasing levels of forest cover. Z-score (color darkness) indicates magnitude of change point along the x axis (how distinctively fish are grouping along the gradient—or magnitude of change). The X axis positioning indicates where along the gradient (at what % forest cover) a significant change point occurs for those specific taxa. Vertical black lines represent median change points for indicator taxa across replicates. The shape of data represents the acceleration of change in Z-scores.


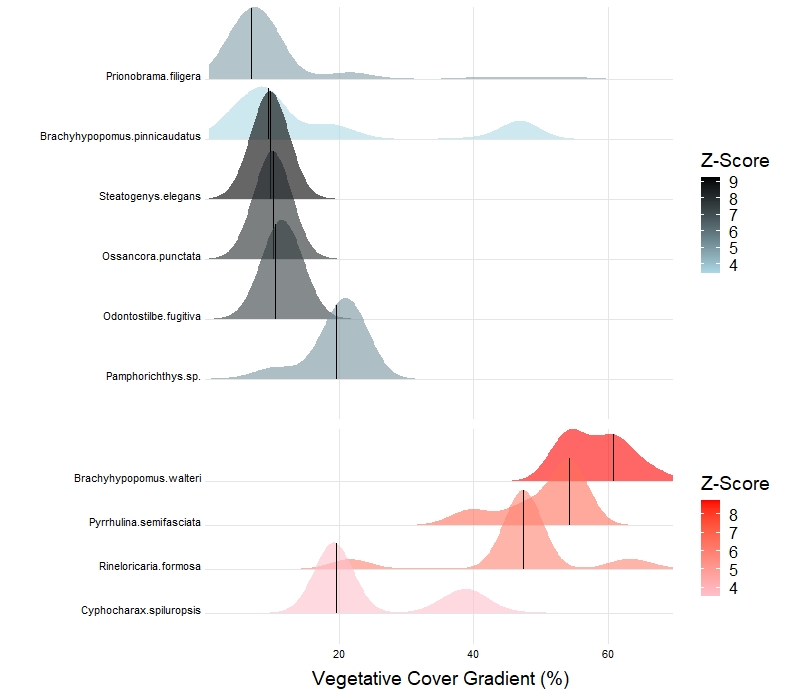


Appendix S9. Pure (> 95% of all 1000 replicates responded the in the same + or – direction to an increase in forest cover) and reliable (bootstrapped environmental change points (CPs) IndVal scores consistently result in p < 0.05) indicator species for a gradient of forest cover identified by TITAN2 throughout the falling water season. Dark values indicate species that decrease with increasing levels of forest cover. Red values indicate species that increase with increasing levels of forest cover. Z-score (color darkness) indicates magnitude of change point along the x axis (how distinctively fish are grouping along the gradient—or magnitude of change). The X axis positioning indicates where along the gradient (at what % forest cover) a significant change point occurs for those specific taxa. Vertical black lines represent median change points for indicator taxa across replicates. The shape of data represents the acceleration of change in Z-scores.
